# Gemcitabine and APG-1252, a novel small molecule inhibitor of BCL-2/BCL-XL, display a synergistic antitumor effect in nasopharyngeal carcinoma through the JAK-2/STAT3/MCL-1 signaling pathway

**DOI:** 10.1038/s41419-021-04042-7

**Published:** 2021-08-05

**Authors:** Fan Luo, Fei-Teng Lu, Miao-Zhen Qiu, Ting Zhou, Wen-Juan Ma, Min Luo, Kang-Mei Zeng, Qiu-Yun Luo, Wen-Tao Pan, Lin Zhang, Zeng-Fei Xia, Zhong-Han Zhang, Jia-Xin Cao, Hong-Yun Zhao, Li Zhang, Da-Jun Yang

**Affiliations:** 1 Department of Experimental Research, State Key Laboratory of Oncology in South China, Collaborative Innovation Center for Cancer Medicine, Sun Yat- Sen University Cancer Center, Guangzhou, China; 2 Department of Medical Oncology, State Key Laboratory of Oncology in South China, Collaborative Innovation Center for Cancer Medicine, Sun Yat-Sen University Cancer Center, Guangzhou, China; 3 Department of Intensive Care Unit, State Key Laboratory of Oncology in South China, Collaborative Innovation Center for Cancer Medicine, Sun Yat-Sen University Cancer Center, Guangzhou, China; 4 Ascentage Pharma (Suzhou) Co, Ltd, 218 Xinghu Street, Suzhou, Jiangsu Province China; 5 Department of Clinical Laboratory Medicine, State Key Laboratory of Oncology in South China, Collaborative Innovation Center for Cancer Medicine, Sun Yat- Sen University Cancer Center, Guangzhou, China; 6 Department of Clinical Research, State Key Laboratory of Oncology in South China, Collaborative Innovation Center for Cancer Medicine, Sun Yat- Sen University Cancer Center, Guangzhou, China

**Keywords:** Chemotherapy, Targeted therapies

## Abstract

Advanced nasopharyngeal carcinoma (NPC) has a poor prognosis, with an unfavorable response to palliative chemotherapy. Unfortunately, there are few effective therapeutic regimens. Therefore, we require novel treatment strategies with enhanced efficacy. The present study aimed to investigate the antitumor efficacy of APG-1252-M1, a dual inhibitor of BCL-2/BCL-XL, as a single agent and combined with gemcitabine. We applied various apoptotic assays and used subcutaneous transplanted NPC model to assess the in vitro and in vivo antitumor activity. Moreover, phospho-tyrosine kinase array was used to investigate the combined therapy’s potential synergistic mechanism. In addition, further validation was performed using immunohistochemistry and western blotting. In vitro, we observed that APG-1252-M1 had moderate antitumor activity toward NPC cells; however, it markedly improved gemcitabine’s ability to promote NPC cell apoptosis and suppress invasion, migration, and proliferation. Specifically, APG-1252 plus gemcitabine exhibited even remarkable antitumor activity in vivo. Mechanistically, the drug combination synergistically suppressed NPC by activating caspase-dependent pathways, blocking the phospho (p)-JAK-2/STAT3/MCL-1 signaling pathway, and inhibiting epithelial-mesenchymal transition. In conclusion, the results indicated that the combination of APG-1252 and gemcitabine has synergistic anticancer activities against NPC, providing a promising treatment modality for patients with NPC.

## Introduction

Nasopharyngeal carcinoma (NPC) is the most frequently diagnosed nasopharyngeal cancer, and is highly prevalent in Southeast Asia and Southern China. Of the 87,000 newly diagnosed NPC cases annually, more than 15% are advanced disease at primary diagnosis [1, 2]. The prognosis for patients with recurrent or metastatic NPC is poor, with a median overall survival (OS) of approximately 20 months [3]. Chemotherapy plays a vital role in advanced NPC. Currently, platinum-based treatment has been proven as the basic regimen for palliative care as a first-line treatment [4, 5]. Unfortunately, there is no standard salvage therapy for patients whose tumor progresses after initial platinum-based regimen failure. Therefore, novel therapeutic approaches with better efficacy are required.

The fluorinated pyrimidine nucleoside analog, gemcitabine, has a broad antitumor activity in various solid tumors, including non-small cell lung cancer, pancreatic cancer, breast cancer, and NPC [6, 7]. The landmark GEM20110714 study demonstrated that gemcitabine plus cisplatin (GP) regimen, as the standard first-line treatment, showed a high response rate and long-term efficacy in recurrent or metastatic NPC [8]. Furthermore, several phase II trials suggested that gemcitabine monotherapy has potent efficacy and tolerable toxicities in NPC, with reported response rates ranging from 28 to 44% [9, 10]. The above studies identified that gemcitabine has positive efficacy and favorable toxicities when used in patients with NPC. However, its survival benefit is still unsatisfactory. Besides, current therapeutic strategies are limited because of a lack of biomarkers for patient selection, low efficacy, physiological side effects, and risks of recurrence. Recently, it has been proposed that targeted therapy would be promising for advanced NPC [11–13]. However, only the epidermal growth factor receptor (EGFR) inhibitors cetuximab and nimotuzumab have been recommended by the NCCN [14]. Therefore, there is still a need for biomarker-driven therapy to enhance the efficacy and durable response of gemcitabine in patients with advanced NPC.

Cancer is characterized by apoptosis resistance, leading to tumor development and progression [15]. The BCL-2 apoptosis regulator (BCL-2) and BCL-2 like 1 (BCL-XL) proteins have been proven to be critical apoptosis inhibitors that are closely correlated with cancer chemoresistance by regulating apoptosis, proliferation, differentiation, and tumor progression [16, 17]. The mammalian BCL-2 protein family is characterized by the presence of four clustered homology domains called BCL-2 homology (BH) domains [18]. BH1, BH2, and BH3 form a hydrophobic groove as the surface binding pocket of BCL-2 and N-terminal BH4 stabilizes the structure [19]. To target BCL-2 family proteins in apoptotic pathways, certain small molecule inhibitors have been investigated [20–22]. APG-1252 is a new BCL-2 homology BH3-mimetic that binds specifically to BCL-2 or BCL-XL’s hydrophobic pocket, and changes to its reactive metabolite, named APG-1252-M1, in vivo can disrupt the anti-apoptotic function of these proteins with potent antitumor effects [23]. Previous studies showed that APG-1252 is active against several types of tumor, such as small cell lung cancer (SCLC), lymphoma, gastric cancer (GC), and acute myeloid leukemia (AML) [23–25]. Currently, to assess its efficacy against cancer, five clinical trials are underway with APG-1252 as a single agent or in combination with chemotherapeutic drugs (NCT03387332, NCT04354727, NCT04001777, NCT04210037, and NCT03080311).

Until now, whether APG-1252 is active against NPC and could promote the antitumor activity of gemcitabine has remained unknown. In the current study, we used various in vitro and in vivo assays to study APG-1252’s preclinical efficacy, either as a solo agent or combined with gemcitabine. The findings demonstrated that APG-1252 combined with gemcitabine had a synergistic effect in the treatment of NPC, and thus represented a promising strategy to improve the therapeutic response of patients with NPC.

## Results

### APG-1252-M1 has anti-NPC activity and exhibits synergy with gemcitabine

**Fig. 1:**
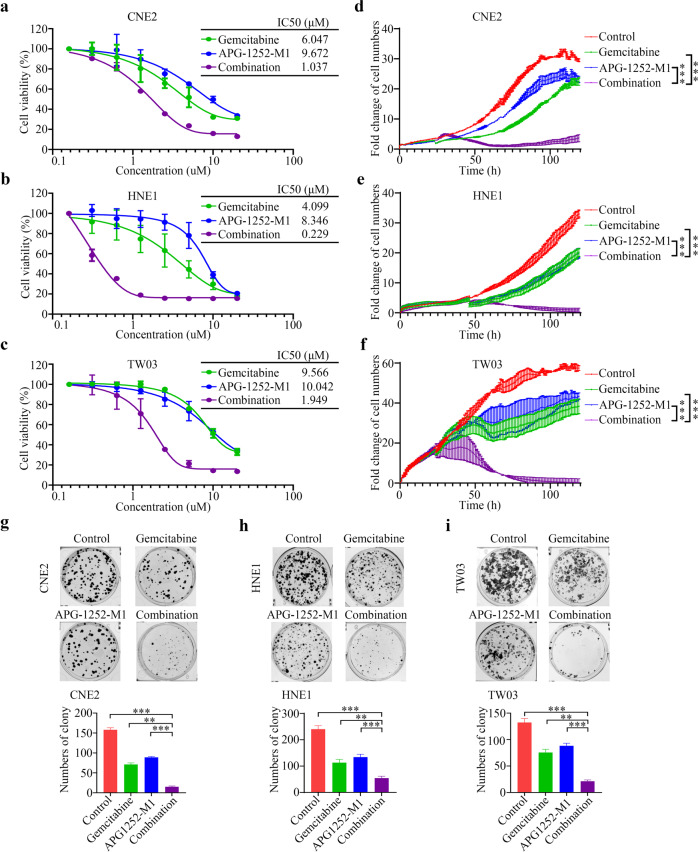
APG-1252-M1 and gemcitabine inhibit the proliferation and oncogenic growth of NPC cells synergistically. **a**–**c** Various concentrations of gemcitabine, APG-1252, or their combination were used to treat CNE2, HNE1, and TW03 cells for 72 h. Cell proliferation was determined using a CCK-8 assay in triplicate. The IC50 values of gemcitabine, APG-1252, and their combination treatment in NPC cells were also analyzed. **d**–**f** Gemcitabine and APG-1252, alone or in combination, were used to treat CNE2, HNE1, and TW03 cells for different times. NPC cell proliferation was detected using RTCA. **g**–**i** The combination of gemcitabine and APG-1252 showed enhanced inhibition of NPC cell colony formation. Gemcitabine, APG-1252 or their combination were used to treat CNE2, HNE1, and TW03 cells at the indicated concentrations for approximately 14 days. The number of colonies stained by crystal violet was used to determine the anti-proliferative effects. The results shown are representative of three independent experiments. All data are presented as the mean±SD. *P < 0.05, **P < 0.01, ***P < 0.001. NPC, nasopharyngeal carcinoma; CCK-8, Cell Counting Kit-8; RTCA, real-time cell analysis.

The novel BCL-2/BCL-XL inhibitor, APG-1252, can bind to BCL-XL and BCL-2 with high affinity. We investigated its therapeutic potential toward NPC by studying the effect of APG-1252-M1 in CNE2, HNE1, TW03 cells, three commonly used NPC cell lines. APG-1252-M1 exhibited excellent anticancer activity, as measured by the CCK-8 assay, with 50% inhibitory concentration (IC50) values of 9.672, 8.346, and 10.042 μM, respectively, which compared slightly above with those of gemcitabine (6.047, 4.099 and 9.566 μM for CNE2, HNE1 and TW03 cells, respectively; Fig. 1a–c). Furthermore, we assessed the activity of APG-1252-M1 combined with gemcitabine. The results showed that APG-1252-M1 significantly enhanced gemcitabine’s anti-NPC activity in a dose-dependent manner (Supplementary Fig. 1a–c). Moreover, the Chou–Talalay method in the Calcusyn software (Biosoft) was used to evaluate their synergistic indexes, which produced a CI value < 0.9, indicating potent synergy between the activities of APG-1252-M1 and gemcitabine (Table 1).

**Table 1:** The CI calculated using the CalcuSyn software.

APG-1252 (μM)	Gemcitabine (μM)	CNE2		HNE1		TW03	
		Fa	CI	Fa	CI	Fa	CI
0.3125	0.3125	0.177	1.006	0.214	0.180	0.113	2.109
0.625	0.625	0.250	1.004	0.364	0.137	0.187	1.410
1.25	1.25	0.369	0.824	0.628	0.068	0.332	0.677
2.5	2.5	0.633	0.298	0.775	0.055	0.664	0.105
5	5	0.785	0.182	0.879	0.043	0.800	0.056
10	10	0.849	0.182	0.899	0.066	0.864	0.048
20	20	0.857	0.328	0.900	0.130	0.872	0.085

*CI* combination index, *CNE2, HNE1, TW03* human cell lines, *Fa* fraction affected.

To evaluate the anti-proliferation activity of the APG-1252-M1-gemcitabine combination further, we explored their effects on CNE2, HNE1, and TW03 cells over time using RTCA. Gemcitabine plus APG-1252-M1 significantly suppressed NPC cell growth in a more potent and durable manner compared with either drug alone (*P* < 0.05; Fig. 1d–f). In the colony formation assay, the application of gemcitabine or APG-1252-M1 individually resulted in only moderate suppression of colony formation. Contrastingly, in combination, the drugs reduced the number of colonies formed to a significantly larger extent (*P* < 0.05; Fig. 1g–i). These observations revealed that APG-1252-M1 has antitumor activity against NPC and could improve the therapeutic effect of gemcitabine significantly.

### APG-1252-M1 enhances the apoptotic effect of gemcitabine in NPC

**Fig. 2:**
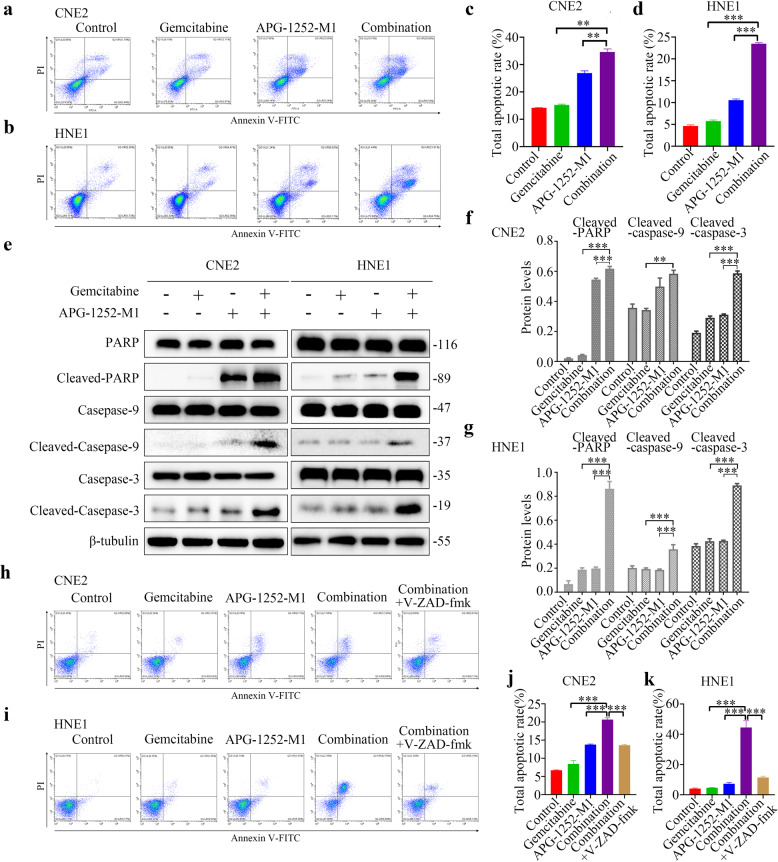
APG-1252-M1 and gemcitabine depend on caspase to induce NPC cell apoptosis. **a**–**d** Annexin V/PI analysis of CNE2 and HNE1 cells following 24 h of exposure to different treatments. The experiments were conducted in triplicate, and the data are shown as the mean±SD. *P < 0.05, **P < 0.01, ***P < 0.001. **e**–**g** Western blotting was applied to detect cleaved PARP, cleaved caspase-3, and cleaved caspase-9 levels as indicators of apoptotic cell death. β-tubulin was used as a loading control. **h**–**k** Quantification of apoptotic CNE2 and HNE1 cells. Annexin V/PI analysis of CNE2 and HNE1 cells following 24 h of exposure to different treatments, including APG-1252-M1 and gemcitabine, or z-VAD-fmk in combination with the two drugs. The experiments were conducted in triplicate, and the data are shown as the mean±SD. *P < 0.05, **P < 0.01, ***P < 0.001. *P < 0.05, **P < 0.01, ***P < 0.001. Error bars indicate the standard deviation of three independently performed experiments. NPC nasopharyngeal carcinoma, *PI* propidium iodide, *PARP* poly (ADP-ribose) polymerase.

The induction of cancer cell apoptosis is a significant anticancer mechanism. Therefore, we sought to determine if APG-1252-M1 and gemcitabine have this therapeutic potential. To that end, we applied Annexin-V staining to evaluate the apoptosis of CNE2, HNE1, and TW03 cells after treatment using APG-1252-M1 and gemcitabine, alone or in combination. Both APG-1252-M1 and gemcitabine resulted in significant apoptosis of tumor cells after treatment for 24 h, which was further increased when the two drugs were applied together (*P* < 0.05; Fig. 2a–d; Supplementary Fig. 2a, b). We next examined whether the combination of APG-1252-M1 and gemcitabine led to cell death dependent on caspases. Analysis using western blotting indicated that in NPC cells treated with gemcitabine and APG-1252-M1 in combination, increased cleavage of PARP, caspase-3, and caspase-9 occurred compared with that caused by either treatment alone (*P* < 0.05; Fig. 2e–g; Supplementary Fig. 2c, d). We further determined the roles of caspases in the apoptosis induced by the combination therapy. It was observed that the pan-caspase inhibitor z-VAD-fmk remarkably decreased the drug combination-induced apoptosis (Fig. 2h–k; Supplementary Fig. 2e, f) and growth inhibition (Supplementary Fig. 2g–j), demonstrating that this drug combination-induced cancer cell apoptosis in a caspase-dependent manner.

### APG-1252-M1 and gemcitabine suppress NPC cell invasion and migration

**Fig. 3:**
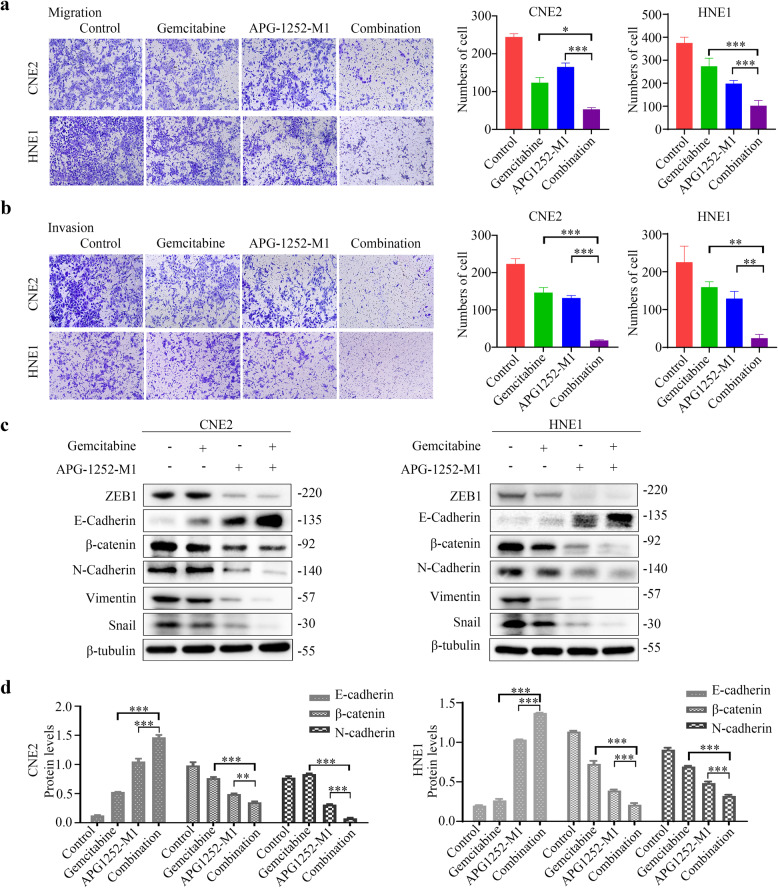
APG-1252-M1 and gemcitabine suppress the biological processes of NPC such as cell migration, invasion, and EMT. **a** CNE2 and HNE1 cells were exposed to different treatments for 24–48 h, including APG-1252-M1, gemcitabine, or in their combination. To evaluate the NPC cells’ migratory ability, Transwell migration assays were carried out. The experiments were conducted in triplicate, and all data are presented as the mean±SD. *P < 0.05, **P < 0.01, ***P < 0.001. **b** CNE2 and HNE1 cells were exposed to different treatments for 24–48 h, including APG-1252-M1, gemcitabine, or their combination. To evaluate the NPC cells’ invasive ability, Transwell invasion assays were carried out. The experiments were performed three times independently, and the data are shown as mean±SD. *P < 0.05, **P < 0.01, ***P < 0.001. **c**, **d** Cells (CNE2 and HNE1) were exposed to different treatments for 24–48 h, including APG-1252-M1, gemcitabine, or their combination. Western blotting was applied to examine the expression of EMT markers (E-cadherin, N-cadherin, β-catenin, ZEB1, Vimentin, and Snail). β-tubulin was used as a loading control. *P < 0.05, **P < 0.01, ***P < 0.001. Error bars indicate the standard deviation of three independently performed experiments. NPC nasopharyngeal carcinoma, EMT epithelial–mesenchyme transition.

NPC metastasis and progression are related to increased tumor cell invasion and motility, which explains the mortality and morbidity of patients with NPC. Next, we evaluated APG-1252-M1 and gemcitabine’s effects on the invasion and migration of NPC cells. Using Transwell assays, we showed that the combined treatment induced a much more potent inhibitory effect on NPC cell migration than that induced by administering either drug alone (*P* < 0.05; Fig. 3a, Supplementary Fig. 3a). APG-1252-M1 and gemcitabine individually only inhibited NPC cell invasion moderately (*P* < 0.05), while the combined treatment suppressed NPC cell invasion to a much greater extent (*P* < 0.05; Fig. 3b, Supplementary Fig. 3b). Collectively, these findings suggested that APG-1252-M1 combined with gemcitabine-induced significantly enhanced anti-metastatic activity against NPC cells.

Epithelial to mesenchymal transition (EMT) is a biological process in which cells convert from an epithelial phenotype to a mesenchymal phenotype, resulting in the loss of cell–cell junctions and enhanced invasion and metastasis [26]. EMT makes a vital contribution to tumor cell metastasis and invasiveness; therefore, we investigated whether APG-1252-M1 and gemcitabine could influence NPC cell EMT. Treatment with either drug alone only resulted in moderate upregulation of the expression of the epithelial marker E-cadherin, whereas it downregulated the levels of the mesenchymal markers β-catenin, N-cadherin, ZEB1, Vimentin, and Snail (Fig. 3c, d; Supplementary Fig. 3c, d). Combined treatment with APG-1252-M1 and gemcitabine inhibited EMT to a much greater extent (Fig. 3c, d; Supplementary Fig. 3c, d). Thus, APG-1252-M1 and gemcitabine demonstrated a marked synergistic effect on the inhibition of NPC cell EMT.

### APG-1252 and gemcitabine vigorously inhibit the tumor growth in vivo

**Fig. 4:**
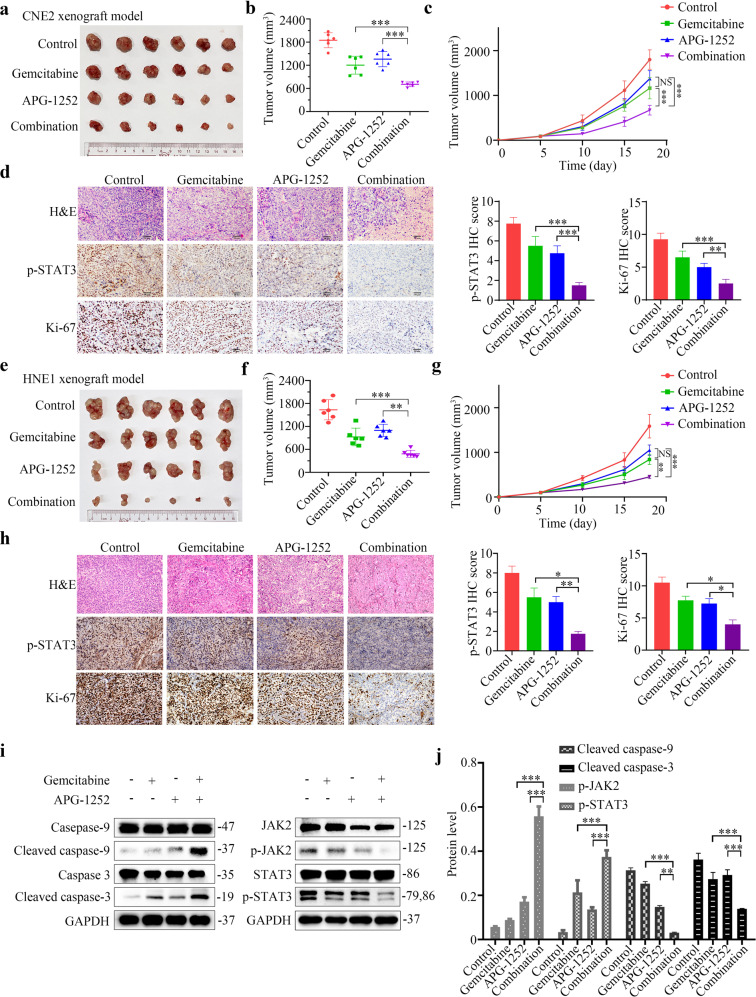
APG-1252 and gemcitabine potently inhibit in vivo NPC tumor growth. **a**, **b**, **e**, **f** Images and volumes of tumors harvested at the end of the experiment; statistical analyses were performed using one-way ANOVA, *P < 0.05, **P < 0.01, ***P < 0.001. **c**, **g** Nude mice with CNE2 and HNE1 xenograft tumors were treated with APG-1252, gemcitabine, alone or in combination, or with the control (saline control). On the indicated days, the tumor volumes were measured (*n* = 6). All data are shown as the mean±SD. Statistical analyses were performed using two-way ANOVA, *P < 0.05, **P < 0.01, ***P < 0.001. **d**, **h** HE and IHC staining were carried out in the indicated xenograft tumors. Data are shown as the mean ± SD of three independently performed experiments. *P < 0.05, **P < 0.01, ***P < 0.001. **i**, **j** Western blotting analysis of the levels of cleaved PARP, cleaved caspase-3, cleaved caspase-9, p-JAK2, and p-STAT3 from HNE1 xenograft tumors from the four different treatment groups. The loading control was β-tubulin. *P < 0.05, **P < 0.01, ***P < 0.001. Error bars indicate the standard deviation of three independently performed experiments. NPC nasopharyngeal carcinoma, ANOVA analysis of variance, HE hematoxylin and eosin, IHC immunohistochemistry, PARP poly (ADP-ribose) polymerase, p-JAK2 phosphorylated Janus kinase 2, p-STAT3 phosphorylated signal transducer and activator of transcription 3.

To examine the in vivo antitumor activity of APG-1252 and gemcitabine, CNE2 and HNE1 xenograft models were established in nude mice. Mice-bearing tumors were divided into four groups randomly, and subjected to treatment for 3 weeks with the vehicle control, gemcitabine, APG-1252, or their combination (*n* = 6 per group). Throughout the experiment, the tumor volume was measured in each group to estimate the antitumor activity. As expected, the antitumor activity of either gemcitabine or APG-1252 alone was low (Fig. 4a–c, e–g). By contrast, the combination of gemcitabine plus APG-1252 generated a much higher anticancer activity compared with that of either drug alone, leading to very little tumor growth (Fig. 4a–c, e–g). Notably, the body weight of all treated mice showed no significant changes compared with that of the untreated animals, suggesting that the combination of APG-1252 and gemcitabine was well-tolerated (Supplementary Fig. 4a, b). In addition, hematoxylin and eosin (HE) staining was performed on the organs of mice under different treatments, such as the brain, heart, lung, stomach, gut, liver, kidney, and spleen to pathologically investigated the toxicity profile, and no obvious structural abnormalities were observed (Supplementary Fig. 4c). To further confirm that APG-1252 plus gemcitabine effectively suppressed tumor growth in a caspase-dependent manner, we collected protein lysates from the tumor tissues of mice harboring HNE1 cell xenografts from various treatments and used western blotting to detect the level of caspase cleavage. The combined treatment increased the cleaved caspase-3 and caspase-9 levels markedly compared with those induced by either treatment alone (Fig. 4i, j). Moreover, we used immunochemistry to evaluate the level of Ki-67 (a proliferation marker) in tumor sections from the experimental mice and observed minimal cell proliferation in the combined treatment group (Fig. 4d, h). These data demonstrated that APG-1252 significantly promoted the anticancer activity of gemcitabine in vivo.

### APG-1252 and gemcitabine trigger synergistic anticancer activity through p-JAK-2/STAT3/MCL-1 signaling

**Fig. 5:**
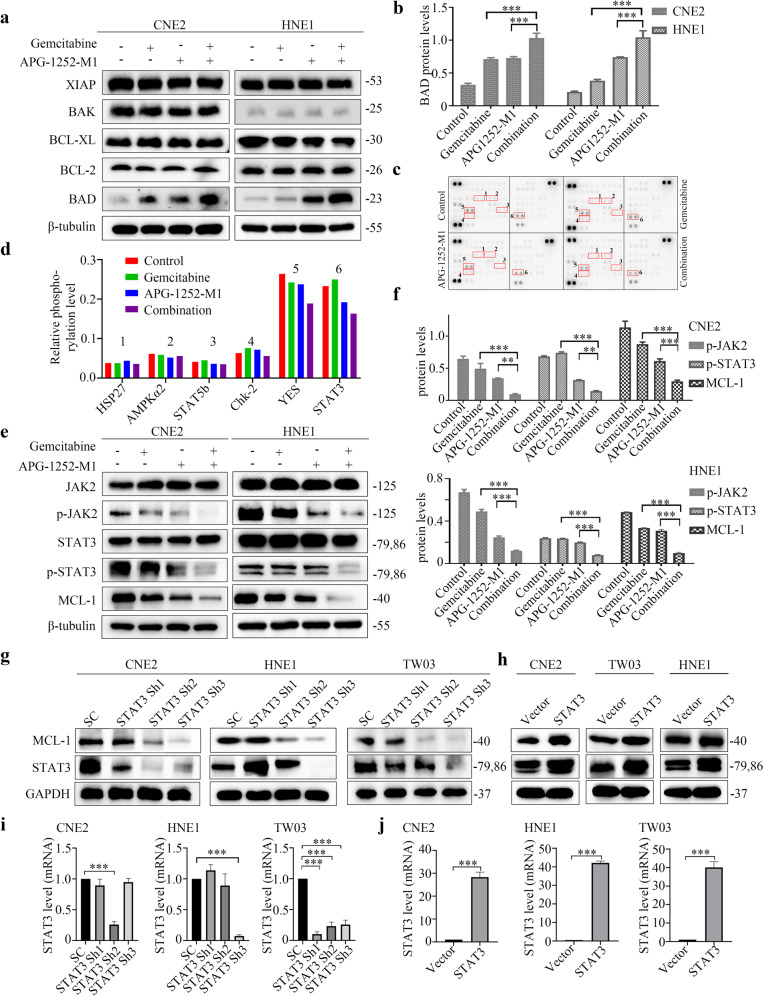
APG-1252-M1 and gemcitabine exhibit a synergistic antitumor effect via inhibiting the JAK2/p-STAT3/MCL-1 signaling pathway. **a**, **b** Western blotting analysis of the levels of BCL-2, BCL-XL, XIAP, BAK, and BAD in the indicated CNE2 and HNE1 cells. The loading control was β-tubulin. *P < 0.05, **P < 0.01, ***P < 0.001. Error bars indicate the standard deviation of three independently performed experiments. **c**, **d** A phospho-kinase array kit was used to analyze the protein lysates of CNE2 cells after different treatments involving control, gemcitabine, APG-1252-M1, and their combination. HSP27, AMPKα2, STAT5b, Chk-2, YES, and STAT3 proteins showed markedly altered levels of phosphorylation (highlighted by red boxes). **e**, **f** Western blotting analysis of the levels of p-JAK-2, JAK-2, p-STAT3(Y705), STAT3, and MCL-1 in the indicated CNE2 and HNE1 cells. The loading control was β-tubulin. *P < 0.05, **P < 0.01, ***P < 0.001. Error bars indicate the standard deviation of three independently performed experiments. **g**, **h** Western blotting assessment of the abundances of STAT3 and MCL-1 in the indicated CNE2, HNE1, and TW03 cells transfected with *STAT3* shRNAs, scrambled control, and STAT3 overexpression vectors. The loading control was GAPDH. **i**, **j** qRT-PCR assessment of the mRNA levels of *STAT3* in the indicated CNE2, HNE1, and TW03 cells transfected with the *STAT3* shRNAs, scrambled control, and STAT3 overexpression vectors. The loading control was *GAPDH*. *P < 0.05, **P < 0.01, ***P < 0.001. Error bars represent the SD of three independent experiments. SC scrambled control, p-JAK2 phosphorylated Janus kinase 2, p-STAT3 phosphorylated signal transducer and activator of transcription 3, BCL-2 BCL-2 apoptosis regulator, BCL-XL BCL-2 like 1, XIAP X-linked inhibitor of apoptosis, BAK BCL-2 homologous antagonist/killer, BAD BCL-2 associated agonist of cell death, MCL-1 MCL-1 apoptosis regulator, BCL-2 family member, HSP27 heat shock protein 27, AMPKα2 protein kinase AMP-activated catalytic subunit alpha 2, STAT5b signal transducer and activator of transcription 5b, Chk-2 checkpoint kinase 2, shRNA short hairpin RNA.

To investigate the mechanism underlying the combined therapy’s induction of tumor apoptosis in NPC, we first detected the effect on the BCL-2 family members. Unexpectedly, the levels of BCL-2 family members, including BCL-2, BCL-XL, BAK, and XIAP did not change significantly; however, the levels of BAD were elevated in CNE2, HNE1, and TW03 cells (Fig. 5a, b; Supplementary Fig. 5a). To further identify the synergistic antitumor mechanism mediated by APG-1252-M1 and gemcitabine, we examined the phosphorylation status of 43 different kinases via human phosphor-kinase analyses (Fig. 5c, d). In the combination group, the phosphorylation of several proteins, especially STAT3(Y705) and YES, was observed to be markedly downregulated. There is currently no antibody to detect specifically the phosphorylated form of YES. Therefore, we can merely use the antibody anti-FYN (phospho Y530) + YES (phospho Y537) to further examined the phosphorylation level of FYN + YES. The verified results of western blotting showed that APG-1252-M1 plus gemcitabine markedly reduced the levels of phosphorylated STAT3 (Y705) in all three NPC cells, while it did not change the levels of p-FYN+p-YES in NPC cells (Fig. 5e, f; Supplementary Fig. 5b). Furthermore, to evaluate which molecule preferentially mediates the downregulation of p-STAT3 in this drug combination, we detected the expression of Janus kinase (JAK) family members simultaneously in NPC cells under different treatments. Consistent with the alteration of p-STAT3, levels of phosphorylated JAK2 were also significantly reduced by the drug combination. We next confirmed the levels of MCL-1 to demonstrate whether the synergistic effect of APG-1252-M1 with gemcitabine was related to STAT3/MCL-1 signaling. We observed that the combination therapy of APG-1252-M1 and gemcitabine significantly reduced the level of p-STAT3(Y705) as well as that of MCL-1 compared with either APG-1252-M1 or gemcitabine alone (Fig. 5e, f; Supplementary Fig. 5c, d). *MCL-1* has been reported as a member of the BCL-2 family with anti-apoptotic characteristics, and inhibition of MCL-1 results in activation of the pro-apoptotic BCL-2 proteins, such as BAD, to induce cell apoptosis [27]. Given that the protein level of BAD was elevated by the combination therapy, we further established stable *BAD* knockdown NPC cells using shRNAs. Among four shRNAs tested, shBAD#1, referred to as BAD sh1, was used in all subsequent experiments (Supplementary Fig. 5e, f). We applied Annexin-V staining to evaluate the apoptosis in scramble control as well as *BAD* knockdown cells treated with APG-1252-M1 and gemcitabine. The results showed that genetic downregulation of *BAD* could partially weaken the synergistic effect of gemcitabine and APG-1252-M, indicating the contribution of BAD to the drug combination-induced apoptosis (Supplementary Fig. 5g, h).

**Fig. 6:**
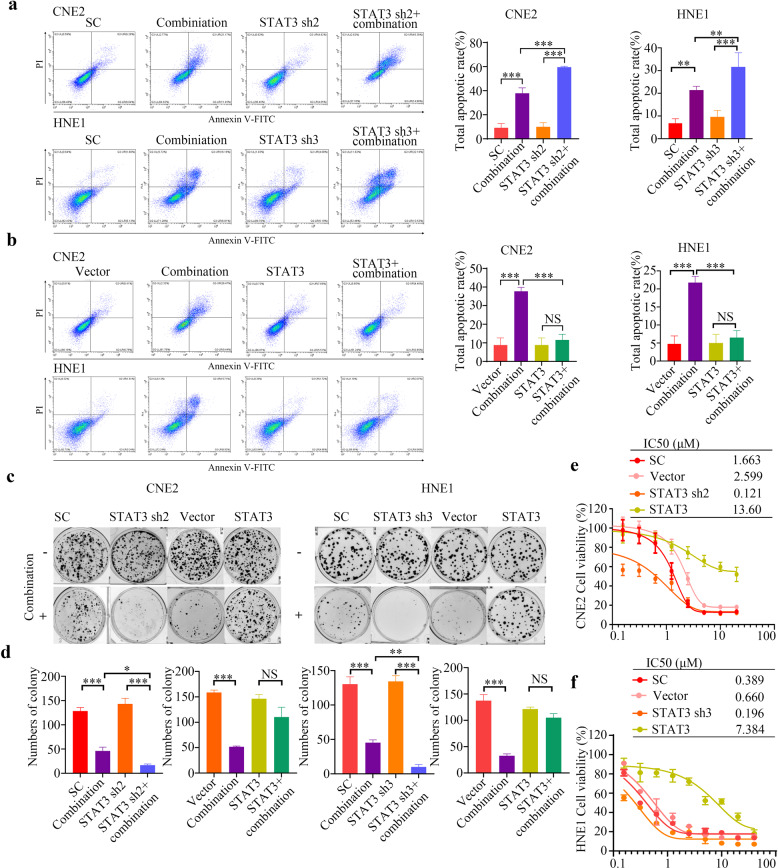
Gene manipulation of STAT3 affects the enhanced antitumor effect of APG-1252 in combination with gemcitabine. **a**, **b** The apoptotic rate of the indicated CNE2, HNE1 cells transfected with the *STAT3* shRNA, scrambled control, and STAT3 overexpression vectors after treatment with or without the combination therapy were determined using an Annexin V/PI assay. *P < 0.05, **P < 0.01, ***P < 0.001. Error bars indicate the standard deviation of three independently performed experiments. **c**, **d** Typical images and enumeration of the indicated cells (scramble control, *STAT3* shRNA, vector, STAT3 overexpression) treated with or without the combination therapy after crystal violet staining. Data are shown as the mean±SD. *P < 0.05, **P < 0.01, ***P < 0.001. **e**, **f** the CCK-8/IC50 values of the indicated CNE2 and HNE1 cells transfection with the *STAT3* shRNA, scrambled control, and STAT3 overexpression vectors after treatment with the combination therapy. *P < 0.05, **P < 0.01, ***P < 0.001. Error bars indicate the standard deviation of three independently performed experiments. SC Scramble control, PI propidium iodide, STAT3 signal transducer and activator of transcription 3, shRNA short hairpin RNA, CCK-8 Cell Counting Kit-8.

STAT3 elevation is critical for cell proliferation. Activated STAT3 becomes dimerized and then translocates to the nucleus, where it binds to specific sites on DNA and upregulates the expression of its target proliferation-associated genes (e.g., those encoding cyclin D1, p21, and c-myc), and pro-survival BCL-2 genes (those encoding BCL-XL, MCL-1, and BCL-2 Like 2 (BCL-W)) [28, 29]. The combined therapy could downregulate MCL-1 levels by suppressing the phosphorylation of STAT3; therefore, we sought to determine whether altering the expression of *STAT3* in NPC cells would affect tumor cell apoptosis induced by APG-1252-M1 plus gemcitabine. We established stable *STAT3* knockdown NPC cells (CNE2, TW03, and HNE1) using shRNAs. Among three shRNAs tested, we used STAT3 Sh2 for CNE2 cell and used STAT3 Sh3 for HNE1 and TW03 cells in subsequent experiments (Fig. 5g, i). Consistent with the aforementioned results, we observed that both the protein level of MCL-1 was potently decreased in *STAT3* knockdown cells (Fig. 5g). Moreover, when shSTAT3 NPC cells were treated with APG-1252-M1 and gemcitabine, colony formation (Fig. 6c, d; Supplementary Fig. 6c, d) and cell proliferation (Fig. 6e, f; Supplementary Fig. 6e) were further inhibited and higher levels of tumor cell apoptosis were observed in comparison with those in the controls (Fig. 6a; Supplementary Fig. 6a). Besides, we transfected NPC cells (CNE2, HNE1, and TW03) with the PCDNA3.1/STAT3 plasmid to overexpress *STAT3* (Fig. 5h, j). Although the combination of APG-1252-M1 and gemcitabine exhibited significant anticancer activity in the empty vector group, involving a reduced number of tumor colonies and cell growth, and increased tumor apoptosis, we observed no significant difference compared with that in the group overexpressing STAT3 (Fig. 6b–f; Supplementary Fig. 6b–e). Further immunohistochemical staining demonstrated that the combined treatment reduced p-STAT3 levels (Fig. 4d, h). These results suggested that the synergistic anticancer activity of APG-1252-M1 combined with gemcitabine functions in a STAT3-dependent manner.

## Discussion

The clinical outcomes for patients with recurrent or metastatic NPC are poor. Chemotherapy has played a significant role in palliative therapy for advanced NPC. Among which platinum-based chemotherapeutic regimens are the most frequently used approaches. However, the serious toxicities of prior platinum-based combination therapy make it difficult for patients who undergo disease progression to receive further treatment [30, 31]. Several phase II and III clinical trials have identified that gemcitabine monotherapy has potent efficacy and favorable toxicities when used in patients with NPC; however, the median survival with single gemcitabine alone in advanced NPC is only 5–16 months [9, 32, 33]. Hence, novel combination approaches are urgently needed to improve the efficacy of gemcitabine. Combined treatment is a valuable strategy to increase antitumor capabilities together with reducing the potential adverse effects resulting from decreasing dosage of the drug when used as a single agent. Furthermore, it might also decrease the occurrence of drug resistance related to the distinct mechanism of each drug’s action. Strategies to boost the anticancer activity of gemcitabine include simultaneously inhibiting the processes of EMT [34], autophagy [35], and cell stemness [36]. In the present study, APG-1252, as a dual inhibitor of BCL-2 and BCL-XL, was employed to enhance gemcitabine’s antitumor activity based on the consideration that overexpression of anti-apoptotic proteins like BCL-2, BCL-XL, and MCL-1, which play a vital role in the process of tumor survival and chemoresistance [37, 38]. The BH3-mimetic APG-1252 is an orally bioavailable BCL-2 family protein inhibitor that binds to anti-apoptotic BCL-2 family proteins BCL-XL and BCL-2 [23]. Our laboratory has reported APG‐1252’s antitumor ability against gastric cancer and AML, and its effect in combination with chemotherapeutic drugs [24, 25]. Previous data demonstrated that APG-1252 had a synergistic effect with the chemotherapeutic drug 5-fluorouracil (5-FU) in gastric cancer cell lines and xenograft animal models via the induction of apoptosis [24]. In addition, several clinical studies are currently underway to test the efficacy of APG-1252 in small cell lung cancer (SCLC) and other advanced cancers (NCT03387332, NCT04354727, NCT04001777, NCT04210037, and NCT03080311). Moreover, it has received approval of FDA as an orphan drug for SCLC. The present study demonstrated that, in vitro and in vivo, APG-1252 and gemcitabine demonstrated marked synergistic activity against NPC. This combined treatment approach might be further developed to produce a new therapy for NPC, and perhaps other solid tumors.

The JAK/STAT signaling pathway is responsible for transmitting the extracellular information from the cell membrane into the nucleus to trigger DNA transcription, and mediates various important physiological functions including cell proliferation, survival, differentiation, apoptosis, and metastasis [39, 40]. In the signaling pathway, JAK2, an important JAKs family member, functions upstream, and is the major activator, of STAT3 [41]. Persistent STAT3 activation is associated with tumor progression of head and neck cancer, NPC, prostate cancer, and lung cancer, implying that phosphorylation of STAT3 is a favorable marker of prognosis of various types of cancer [42–44]. Besides, constitutively activated STAT3 can also upregulate the expression of anti‐apoptotic genes, including *BCL-XL* and *MCL-1*, in human cancer cells [45]. However, there is also study showing that STAT3 inhibition reduces the MCL-1 expression while not changes the protein level of BCL-XL [46], which is consistent with our findings. Zhou et al. reported that STAT3 could regulate *MCL-1* at the transcriptional level in lung cancer cell lines [47]. The anti-apoptotic protein and member of the BCL-2 family, MCL-1, is regulated by several growth factors [48]. Furthermore, MCL-1 overexpression has been observed in various tumors, and is associated with escape from cell apoptosis and acquisition of chemoresistance [49]. Yang et al. revealed that the BCL-2 inhibitor TW-37 could enhance chemosensitivity by inhibiting STAT3/MCL-1 signaling [50]. Next, we questioned whether the synergy between APG-1252 and gemcitabine exhibited in NPC by regulating the JAK-2/p-STAT3/MCL-1 pathway. Consequently, we performed phospho-tyrosine kinase array and RTK array experiments, as well as western blotting validation, to explore the underlying mechanism. In line with the evidence that phosphorylated STAT3 lies upstream of MCL-1 signaling, our study showed that the combined treatment of gemcitabine and APG-1252 led to downregulation of JAK-2/p-STAT3/MCL-1 signaling, together with activation of the apoptotic cascade, consisting of caspases and the pro-apoptotic protein BAD, in NPC cells. Previous studies demonstrated BAD binds to and neutralizes anti-apoptotic proteins BCL-2, BCL-XL, and BCL-W, would make for a diversion of BAX proteins from BCL/BCL-XL oligomerization and promoting cell death [51–53]. Meanwhile, MCL-1 is a critical apoptosis-regulatory molecule, whose reduced expression in cooperation with BAD induces apoptosis [27].

The combined therapy attenuated MCL-1 expression dependent on interrupting STAT3 phosphorylation. In addition, previous studies showed that pharmacological or genetic interruption of STAT3 could enhance anti-NPC activity [50, 54]. Hence, we further established both *STAT3* knockdown and overexpression models in vitro to determine whether STAT3 could influence the NPC cell apoptosis caused by the combined therapy. We demonstrated that knockdown of *STAT3* reduced MCL-1 expression and augmented the cell death induced by APG-1252 plus gemcitabine, while *STAT3* overexpression weakened the synergistic antitumor effect of APG-1252 and gemcitabine. Thus, our data revealed that the cooperative antitumor effect of this combined treatment is probably dependent on the suppression of STAT3 signaling.

Recent research has suggested that EMT has an important function in tumor metastasis and contributes to resistance to both radiotherapy and chemotherapy in NPC [30, 55]. Notably, NPC is invariably associated with EBV infection. Increasing evidence indicates that EBV-encoded oncoproteins, such as latent membrane protein 1 (LMP1) activate several intracellular signaling pathways, including JAK2/STAT3, phosphatidylinositol-4,5-bisphosphate 3-kinase (PI3K)/protein kinase B (AKT), and mitogen activated protein kinase (MAPK) pathways [56, 57]. All of these signaling pathways could deliver signals that promote EMT in NPC [58]. The combined therapy of gemcitabine and APG-1252 suppressed NPC cell migration and invasion to a greater extent than either drug alone, demonstrating that they are particularly effective to treat of metastatic and invasive advanced NPC. Under normal conditions, cell-cell association is based on a complex formed between β-catenin and E-cadherin. Here, the combination therapy reduced EMT, as indicated by the markedly decreased N-cadherin and β-catenin levels and increased E-cadherin levels. The combination of gemcitabine and APG-1252 potently restrained EMT, which was in accordance with their inhibitory action on NPC cell migration and invasion.

## Conclusion

**Fig. 7:**
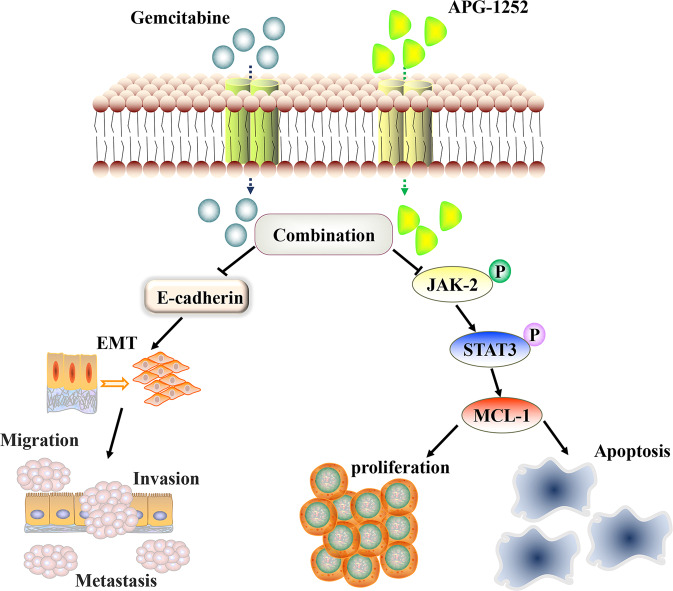
The inhibitory mechanism of APG-1252 and gemcitabine against nasopharyngeal carcinoma. APG-1252 plus gemcitabine synergistically suppressed NPC by activating caspase-dependent pathways, blocking the phosphor (p)JAK2/STAT3/MCL-1 signaling pathway, and inhibiting EMT process.

The results of the present study revealed that a combination of APG-1252 and gemcitabine synergistically inhibited the in vitro and in vivo invasion, migration, and proliferation of NPC cells. Furthermore, they induced NPC cells to undergo apoptosis by inactivating the JAK-2/STAT3/MCL-1 signaling pathway (Fig. 7). These observations identified a potential strategy to treat NPC and will be pivotal for translation into the clinic in the future, with the aim of reducing the mortality and morbidity of patients with NPC.

## Methods

### Cells culture and regents

The present study used the human NPC cell lines CNE2, HNE1, and TW03. Professor Kaitai Yao (Southern Medical University, Guangzhou, People’s Republic of China) kindly donated the HNE1 cells. Professor Musheng Zeng (Sun Yat-Sen University Cancer Center, Guangzhou, People’s Republic of China) kindly provided the CNE2 and TW03 cells. Dulbecco’s modified Eagle’s medium (DMEM) (Gibco Life Technologies, Grand Island, NY, USA) containing 10% fetal bovine serum (FBS) (Gibco Life Technologies) and 1% Penicillin-Streptomycin (Thermo Fisher Scientific, Waltham, MA, USA) was used to culture all NPC cells in a humidified incubator containing 5% CO_2_ at 37 °C. APG-1252 and APG-1252-M1 were purchased from Ascentage Pharma Group Inc (Jiangsu, China). For the in vitro experiments APG-1252-M1 was dissolved in dimethyl sulfoxide (DMSO) at 10 μM and kept at −20 °C. For the in vivo experiments, APG-1252 was dissolved in 10% polyethylene glycol 4000 (PEG400)/5% Castor oil ethoxylated (EL)/85% phosphate-buffered saline (PBS). Gemcitabine and z-VAD-fmk were purchased from Selleck Chemicals (Houston, TX, USA), and were prepared as a stock concentration at 10 mM in DMSO and stored at −20 °C.

### Western blotting

Cells were treated with various concentrations of the drugs (detailed in the figures). After 48 h, the cells were harvested and washed twice with cold PBS. Cell Lysis Buffer (Cell Signaling Technology, Danvers, MA, USA) was used to lyse the cells, and a bicinchoninic acid (BCA) Protein Assay Kit (Thermo Fisher Scientific) was used to measure the protein concentration of the lysates. Electrophoresis using 8–15% SDS-PAGE was used to separate the total proteins, which were then electrotransferred to polyvinylidene fluoride (PVDF) membranes (Roche, Basel, Switzerland). 5% non-fat milk was used to block the membranes, which were then incubated with primary antibodies (1:1000) recognizing STAT3, p-STAT3, Janus kinase 2 (JAK-2), phosphor (p)-JAK-2, BCL-2, BCL-XL, BCL-2 associated agonist of cell death (BAD), MCL-1 apoptosis regulator BCL-2 family member (MCL-1), X-linked inhibitor of apoptosis (XIAP), BCL-2 antagonist/killer 1 (BAK), Caspase-3, Cleaved Caspase-3, Caspase-9, poly(ADP-ribose) polymerase (PARP), E-cadherin, N-cadherin, β-catenin, Zinc Finger E-Box Binding Homeobox 1 (ZEB1), Vimentin, Snail, YES, Anti-Fyn (phospho Y530) + Yes (phospho Y537) (Abcam, Cambridge, MA, USA). Glyceraldehyde-3-phosphate dehydrogenase (GAPDH), and β-tubulin (Cell Signaling Technology) were used as controls. Horseradish peroxidase (HRP)-conjugated goat anti-mouse or anti-rabbit antibodies (1:5000; Santa Cruz Biotechnology, Santa Cruz, CA, USA) were used as secondary antibodies. A Bio-Rad Clarity™ western ECL substrate (Bio-Rad Laboratories, Hercules, CA, USA) was used to visualize the immunoreactive protein bands and Image Lab (Bio-Rad) was used to quantify the protein levels.

### Cell viability assays

Cells (3 × 10^3^ cells per well) were cultured in wells of in 96-well plates containing 200 μL of culture medium for 12 h. After the cells had adhered, they were pretreated with the indicated concentrations of APG-1252-M1, gemcitabine, or their combination for 72 h, respectively. Then, the Cell Counting Kit-8 (CCK-8; 20 μL) reagent (Dojindo Laboratories, Kumamoto, Japan) was added to 100 μL of culture medium per well and incubated for 2–4 h at 37 °C. The absorbance value was then measured using a spectrophotometer at 450 nm. All experiments were performed in triplicate per trial and conducted at least three times. Nonlinear regression in GraphPad Prism version 8.0 (GraphPad Software Inc., La Jolla, CA, USA) was used to analyze the half maximal inhibitory concentration (IC50) values and dose-response curves. Moreover, drug-combination effects were analyzed using CalcuSyn software (Biosoft, Cambridge, UK) to calculate the combination index (CI) for each concentration tested, whereby CI values < 0.9, = 0.9, and > 0.9 indicate synergy, additivity, and antagonism, respectively.

### Assay of colony formation

NPC cells (2 × 10^3^ cells per well) were cultured in 6-well plates for 12 h before being added with the indicated nominal concentrations of drugs. Ten or 14 days later, the colonies were fixed using 4% paraformaldehyde for 10 min and stained using crystal violet for 30 min, after which the crystal violet was washed out. Finally, an inverted microscope was used to acquire images of the cells in the wells and the observable colonies (those with more than 50 cells) were recorded. In the figures, the presented images are representative of three independent experiments.

### Migration and invasion assays

CNE2, HNE1 and TW03 cells (5 × 10^4^ cells in serum-free DMEM (200 μL)) were added to the top chamber of a 24-well Transwell plate (Corning, NY, USA), and treated with control (PBS), APG-1252-M1, gemcitabine, or their combination, respectively. For invasion assays, the upper chamber membranes were coated with Matrigel (Corning). Then, 700 μL of DMEM with 20% FBS were placed in the lower chamber and cultured in 5% CO_2_ at 37 °C for 18–36 h. Methanol was then used to fix the cells for 10 min, after which 1% crystal violet staining was carried out for 30 min at room temperature. The cells were counted under a microscope in five randomly chosen fields per well. Each assay was carried out independently three times.

### Real-time cell analysis

Real-Time Cell Analysis (RTCA) was conducted using the RTCA S16 (ACEA Biosciences, San Diego, CA, USA) to monitor the process of cell proliferation. The microelectrodes were attached at the bottom of the modified 16-well E-plates (Roche Diagnostics GmbH, Mannheim, Germany) for impedance-based detection during cell attachment, and the proliferation of the cells was indicated by the cell index value. The cell suspension (200 μL) was plated in the 16-well E-plates at a density of 1×10^3^ cells/well. The E-plate was first incubated at room temperature for 30 min, and then transferred into the humidified incubator for 12 h until a stable baseline was reached. The cells were then treated as four groups (control, APG-1252-M1, gemcitabine, and their combination) at nominal concentrations, and the cell index recording was continued. The cell index was recorded every 15 min for 120 h to measure cell proliferation inhibition.

### Analysis of apoptosis via flow cytometry

An Annexin V-propidium iodide (PI) apoptosis detection kit (BD Biosciences, San Jose, CA, USA) was used to determine cell apoptosis via flow cytometry (Beckman Coulter, Indianapolis, IN, USA). Cells (2 × 10^6^ per well) were cultured in 6-well plates before being treated with the indicated drug concentrations for 48 h. After treatment, we harvested the cells, washed them twice with PBS, and stained them using Annexin V-fluorescein isothiocyanate (FITC) and PI in the dark following the manufacturer’s protocol, followed by analysis using Beckman flow cytometry. The upper right quadrant represents late apoptotic cells, and the lower right quadrant represents early apoptotic cells. The assessment of the apoptosis rate was the sum of early and late apoptosis.

### Transfection of shRNA and plasmid DNA

According to the manufacturer’s instructions, vectors expressing *STAT3* short hairpin RNAs (shRNAs) and an shRNA scrambled control (Supplementary Table S1) were transiently transfected using a pSIH-H1-puro Lentivector Packaging Kit (System Biosciences, Palo Alto, CA, USA). The *STAT3* cDNA (Supplementary Table S2) was amplified and cloned into the PCDNA3.1 plasmid (Promega, Madison, WI, USA). Transfections of HEK293T cells at approximately 80% confluency were performed in 10 cm dishes with the aid of the Lipofectamine 2000 transfection reagent (Life Technologies) following the manufacturer’s protocols. *BAD* expression was inhibited using shRNAs (HSH015538-31-LVRU6MP for *BAD* shRNA and LPP-CSHCTR001-LVRU6MP for relative scramble control; GeneCopoeia). Fresh growth medium replaced the transfection medium at 5 h after transfection. At 48 h after transfection, we collected the viral supernatant and filtered it through a 0.45-nm filter (System Biosciences). The viral particles were used to infect CNE2, HNE1 and TW03 cells overnight at 37 °C. Forty-eight hours later, the cells were selected using growth medium supplemented with puromycin at 5 μg/mL. The medium was changed every 2 days for 2–3 weeks, until isolated colonies (∼2 mm diameter) formed on the plate. Individual clones were then transferred to 12-well dishes containing 1 μg/mL puromycin and expanded for further analysis. Stable inhibition of *STAT3* and *BAD* expressions were verified using quantitative real-time reverse transcription PCR (qRT-PCR) and western blotting.

### RNA extraction and quantitative real-time reverse transcription PCR

Total cellular RNA was extracted using Trizol (Invitrogen) for mRNA analysis. A Transcriptor First Strand cDNA Synthesis kit (Roche Applied Science, Madison, WI, USA) was used to reverse transcribe the RNA to cDNA. The cDNA was then used as the template in quantitative real-time PCR (qPCR) reactions. The FastStart SYBR Green Master (ROX) reagent (Roche Applied Science) was used to perform the qPCR reactions following the manufacturer’s protocols. The Bio-Rad CFX96 system with SYBR green (Bio-Rad) and the appropriate primers were used for qPCR to estimate the *STAT3* mRNA expression level. Triplicate data were normalized to the *GAPDH* mRNA levels in the samples. The primers used were as follows: *STAT3* forward: CTTGACACACGGTACCTGGA; reverse: CTTGCAGGAAGCGGCTATAC; *BAD* forward: CCCAGAGTTTGAGCCGAGTG; reverse: CCCATCCCTTCGTCGTCCT; *GAPDH* forward: GGTGAAGGTCGGAGTCAACGG; reverse: CCTGGAAGATGGTGATGGGATT.

### Human phosphor-kinase array

CNE2 cells were placed at 5 × 10^6^ cells per 10 cm dish and four groups were treated with the indicated concentrations of drugs for 48 h. The Human Phospho-Kinase Array kit (R&D Systems, Minneapolis, MN, USA) was used to analyze the cells following the manufacturer’s protocol. Quantity One Software (Bio-Rad) was used to image and quantify the chemiluminescent signals.

### Histology and immunohistochemistry (IHC)

Mouse tumor tissues were fixed with formalin, embedded in paraffin, and cut into slices at 3-μm-thick for IHC analysis, which was carried out in accordance with standard procedures. Primary antibodies recognizing marker of proliferation Ki-67 (Ki-67) (1:500) and p-STAT3 (1:500) (Abcam) were used. The positive signals were captured under a fluorescence microscope.

### In vivo mouse studies

All mice were purchased from Vital River Laboratory Animal Technology Co., Ltd, (Beijing, China), and raised in a specific pathogen-free (SPF) experimental animal room under standard conditions. All animal protocols were approved by the institutional animal care and use committee at Sun Yat-Sen University cancer center. Female BALB/c nude mice (weighing 14–18 g; 6 weeks old) were injected with 5 × 10^6^ CNE2 or HNE1 cells in 200 μL of PBS subcutaneously. The mouse tumor sizes were measured using electronic calipers and estimated using the formula: Tumor volume (cm^3^) = (length × width^2^)/2.

The mice bearing tumors were assigned randomly to four groups when their tumor sizes increased to ~200 mm^3^: Control group, APG-1252 group, gemcitabine group, and the combination of APG-1252 and gemcitabine group (each group, n = 6). For the APG-1252-treated mice, 50 mg/kg of APG-1252 was injected via the caudal vein twice a week. For the gemcitabine-treated mice, 50 mg/kg of gemcitabine was delivered via intraperitoneal injection twice a week. Tumors were measured three times a week using calipers until a terminal event endpoint was reached (tumor volume = 2000 mm^3^). Kaplan–Meier survival curves were created, and among the different groups, the log-rank test was used to assess the statistical differences among the survival curves.

### Statistical analysis

GraphPad Prism statistical analysis software v8.0 used to perform the statistical analysis (two-way analysis of variance (ANOVA), one-way ANOVA, Student’s *t* test). Data were expressed as the mean±standard deviation (SD) from at least three independent experiments. Differences in *P*-values < 0.05 were considered statistically significant.

## Supplementary information

Supplementary Figure and Table legends

Supplymentary Table

Supplementary Figure 1

Supplementary Figure 2

Supplementary Figure 3

Supplementary Figure 4

Supplementary Figure 5

Supplementary Figure 6

## Data Availability

The data generated and analyzed will be made from the corresponding author on reasonable request. The authenticity of this article has been validated by uploading the key raw data onto the Research Data Deposit public platform (www.researchdata.org.cn), with the approval RDD number as RDDB2021001648.
