# Supplementary material for: Gemcitabine and APG-1252, a novel small molecule inhibitor of BCL-2/BCL-XL, display a synergistic antitumor effect in nasopharyngeal carcinoma through the JAK-2/STAT3/MCL-1 signaling pathway
Source: Cell Death Dis. 2021 Aug 5;12(8):772. doi: 10.1038/s41419-021-04042-7 (PMC8342713; doi:10.1038/s41419-021-04042-7)
Supplement: Supplementary file 1 — Supplementary Figure and Table legends [file 41419_2021_4042_MOESM1_ESM.docx]

**Supplementary Figure and Table legends**

**Supplementary Figure 1: APG-1252-M1 and gemcitabine inhibit the proliferation and oncogenic growth of NPC cells synergistically**. (a-c) Various concentrations of gemcitabine, APG-1252-M1, or their combination were used to treat CNE2, HNE1, and TW03 cells for 72 h. Histogram and bar chart showing the cell proliferation rate.

**Supplementary Figure 2: APG-1252-M1 and gemcitabine depend on caspase to induce NPC cell apoptosis**. (a,b) Annexin V/PI analysis of TW03 cells following 24 h of exposure to different treatments. The experiments were conducted in triplicate, and the data are shown as the mean ± SD. * P < 0.05, ** P < 0.01, *** P < 0.001. (c,d) Western blotting was applied to detect cleaved PARP, cleaved caspase-3, and cleaved caspase-9 levels as indicators of apoptotic cell death. β-tubulin was used as a loading control. * P < 0.05, ** P < 0.01, *** P < 0.001. Error bars indicate the standard deviation of three independently performed experiments. (e,f) Annexin V/PI analysis of TW03 cells following 24 h of exposure to different treatments (g-j) Colony formation analysis of NPC cells that were treated with gemcitabine, APG-1252-M1, the drug combination with or without z‐VAD‐fmk (14 d after cells plating). The number of colonies stained by crystal violet was used to determine the anti-proliferative effects. The results shown are representative of three independent experiments. All data are presented as the mean ± SD. * P < 0.05, ** P < 0.01, *** P < 0.001.

**Supplementary Figure 3: APG-1252-M1 and gemcitabine suppress the biological processes of NPC such as cell migration, invasion, and EMT.** (a) TW03 cells were exposed to different treatments for 24–48 h, including APG-1252-M1, gemcitabine, or in their combination. To evaluate the NPC cells’ migratory ability, Transwell migration assays were carried out. The experiments were conducted in triplicate, and all data are presented as the mean ± SD. * P < 0.05, ** P < 0.01, *** P < 0.001. (b) TW03 cells were exposed to different treatments for 24–48 h, including APG-1252-M1, gemcitabine, or their combination. To evaluate the NPC cells’ invasive ability, Transwell invasion assays were carried out. The experiments were performed three times independently, and the data are shown as mean ± SD. * P < 0.05, ** P < 0.01, *** P < 0.001. (c) TW03 cells were exposed to different treatments for 24–48 h, including APG-1252-M1, gemcitabine, or their combination. Western blotting was applied to examine the expression of EMT markers (E-cadherin, N-cadherin, β-catenin, ZEB1, Vimentin and Snail). β-tubulin was used as a loading control. * P < 0.05, ** P < 0.01, *** P < 0.001. Error bars indicate the standard deviation of three independently performed experiments.

**Supplementary Figure 4: APG-1252 and gemcitabine potently inhibit in vivo NPC tumor growth.** (a,b) The body weight of the CNE2 and HNE1 xenograft mice in different groups was recorded. Data are shown as the mean ± SD. (c) HE staining for brain, heart, lung, stomach, gut, liver, kidney, spleen was carried out in the indicated mice treated with APG-1252 and gemcitabine, alone or in combination.

**Supplementary Figure 5: APG-1252-M1 and gemcitabine exhibit a synergistic anti-tumor effect via inhibiting the JAK2/p-STAT3/MCL-1 signaling pathway**. (a) Western blotting analysis of the levels of BCL-2, BCL-XL, XIAP, BAK and BAD in the indicated TW03 cells. The loading control was β-tubulin. * P < 0.05, ** P < 0.01, *** P < 0.001. Error bars indicate the standard deviation of three independently performed experiments. (b) Western blotting analysis of the levels of p-FYN+p-YES and YES in the indicated CNE2, HNE1 and TW03 cells. The loading control was β-tubulin. (c,d) Western blotting analysis of the levels of p-JAK2, JAK2, p-STAT3(Y705), STAT3, and MCL-1 in the indicated TW03 cells. The loading control was β-tubulin. * P < 0.05, ** P < 0.01, *** P < 0.001. Error bars indicate the standard deviation of three independently performed experiments. * P < 0.05, ** P < 0.01, *** P < 0.001. Error bars represent the SD of three independent experiments. (e) Western blotting assessment of the abundances of BAD in the indicated CNE2, HNE1 and TW03 cells transfected with *BAD* shRNAs and scrambled control. The loading control was GAPDH. (f) qRT-PCR assessment of the mRNA levels of *BAD* in the indicated CNE2, HNE1, and TW03 cells transfected with the *BAD* shRNAs and scrambled control. The loading control was *GAPDH*. (g,h) Annexin V/PI analysis of CNE2, HNE1 and TW03 cells and shBAD cells following 24 h of exposure to different treatments. The experiments were conducted in triplicate, and the data are shown as the mean ± SD. * P < 0.05, ** P < 0.01, *** P < 0.001.

**Supplementary Figure 6:** **Gene manipulation of STAT3 affects the enhanced anti-tumor effect of APG-1252 in combination with gemcitabine.** (a,b) The apoptotic rate of the indicated TW03 cells transfected with the STAT3 shRNA, scrambled control, and STAT3 overexpression vectors after treatment with or without the combination therapy were determined using an Annexin V/PI assay. * P < 0.05, ** P < 0.01, *** P < 0.001. Error bars indicate the standard deviation of three independently performed experiments. (c,d) Typical images and enumeration of the indicated TW03 cells (scramble control, STAT3 shRNA, vector, STAT3 overexpression) treated with or without the combination therapy after crystal violet staining. Data are shown as the mean ± SD. * P < 0.05, ** P < 0.01, *** P < 0.001. (e) the CCK-8/IC50 values of the indicated TW03 cells transfection with the STAT3 shRNA, scrambled control, and STAT3 overexpression vectors after treatment with the combination therapy. * P < 0.05, ** P < 0.01, *** P < 0.001. Error bars indicate the standard deviation of three independently performed experiments.

**Supplementary table S1. STAT3 shRNAs for vectors
Supplementary table S2. Primers for vector construction.**
