# Supplementary material for: Gemcitabine and APG-1252, a novel small molecule inhibitor of BCL-2/BCL-XL, display a synergistic antitumor effect in nasopharyngeal carcinoma through the JAK-2/STAT3/MCL-1 signaling pathway
Source: Cell Death Dis. 2021 Aug 5;12(8):772. doi: 10.1038/s41419-021-04042-7 (PMC8342713; doi:10.1038/s41419-021-04042-7)
Supplement: Supplementary file 2 — Supplymentary Table [file 41419_2021_4042_MOESM2_ESM.docx]

**Supplementary table S1. shRNAs for vectors**

| Gene | shRNA |
| --- | --- |
| Scramble control | CAACAAGATGAAGAGCACCAA |
| Stat3sh1 | TCTCTGCAGAATTCAA |
| Stat3sh2 | CAGGCTGGTAATTTATATAAT |
| Stat3sh3 | GGCGTCCAGTTCACTACTA |

**Supplementary table S2. Primers for vector construction.**

|  | Forward | Reverse |
| --- | --- | --- |
| Stat3/pCDNA3.1 vector | ATGGCCCAATGGAATCAGC | TCACATGGGGGAGGTAGCG |
