# Supplementary figures and images for: Gemcitabine and APG-1252, a novel small molecule inhibitor of BCL-2/BCL-XL, display a synergistic antitumor effect in nasopharyngeal carcinoma through the JAK-2/STAT3/MCL-1 signaling pathway
Source: Cell Death Dis. 2021 Aug 5;12(8):772. doi: 10.1038/s41419-021-04042-7 (PMC8342713; doi:10.1038/s41419-021-04042-7)

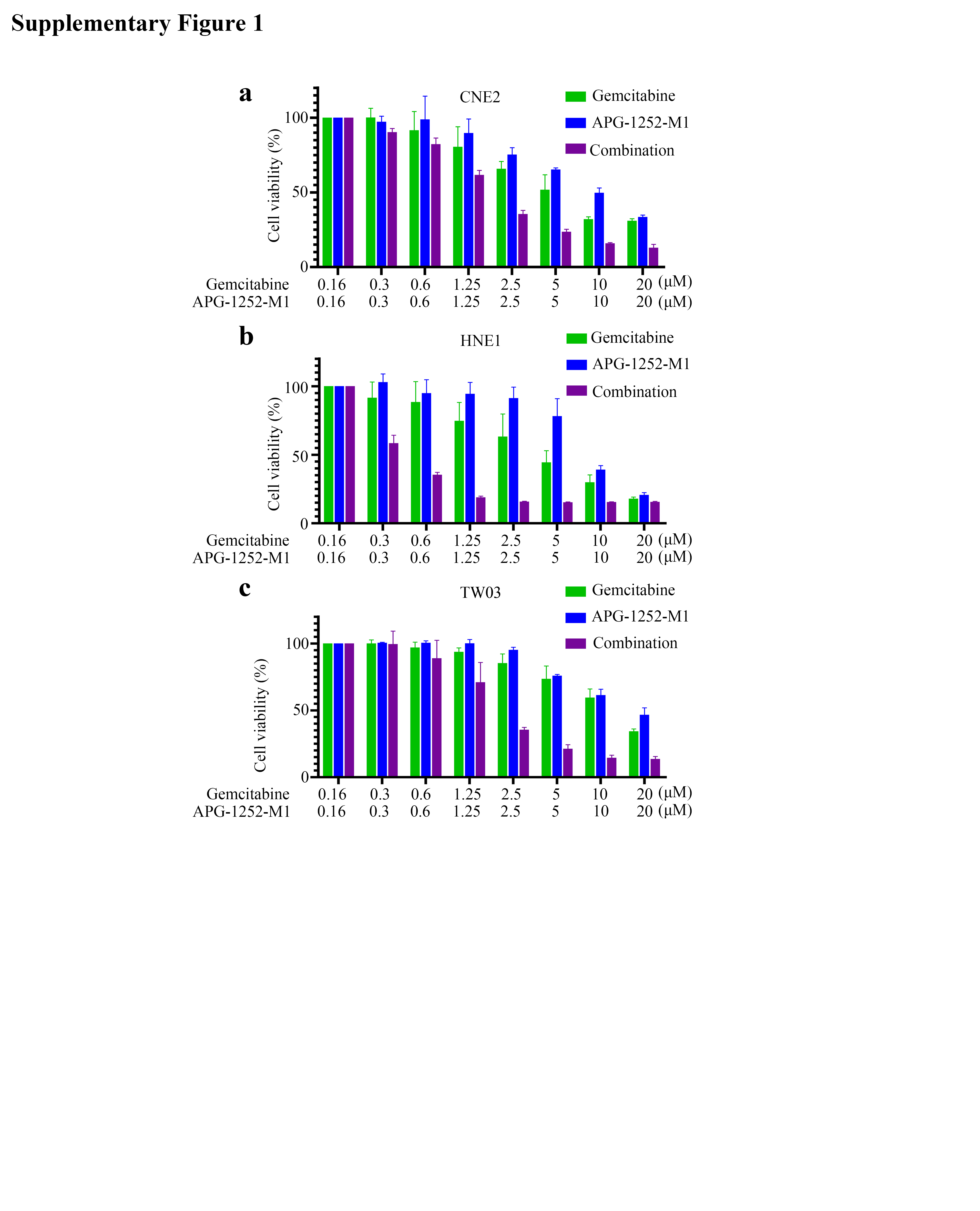

Supplement: Supplementary file 3 — Supplementary Figure 1 [file 41419_2021_4042_MOESM3_ESM.jpg]

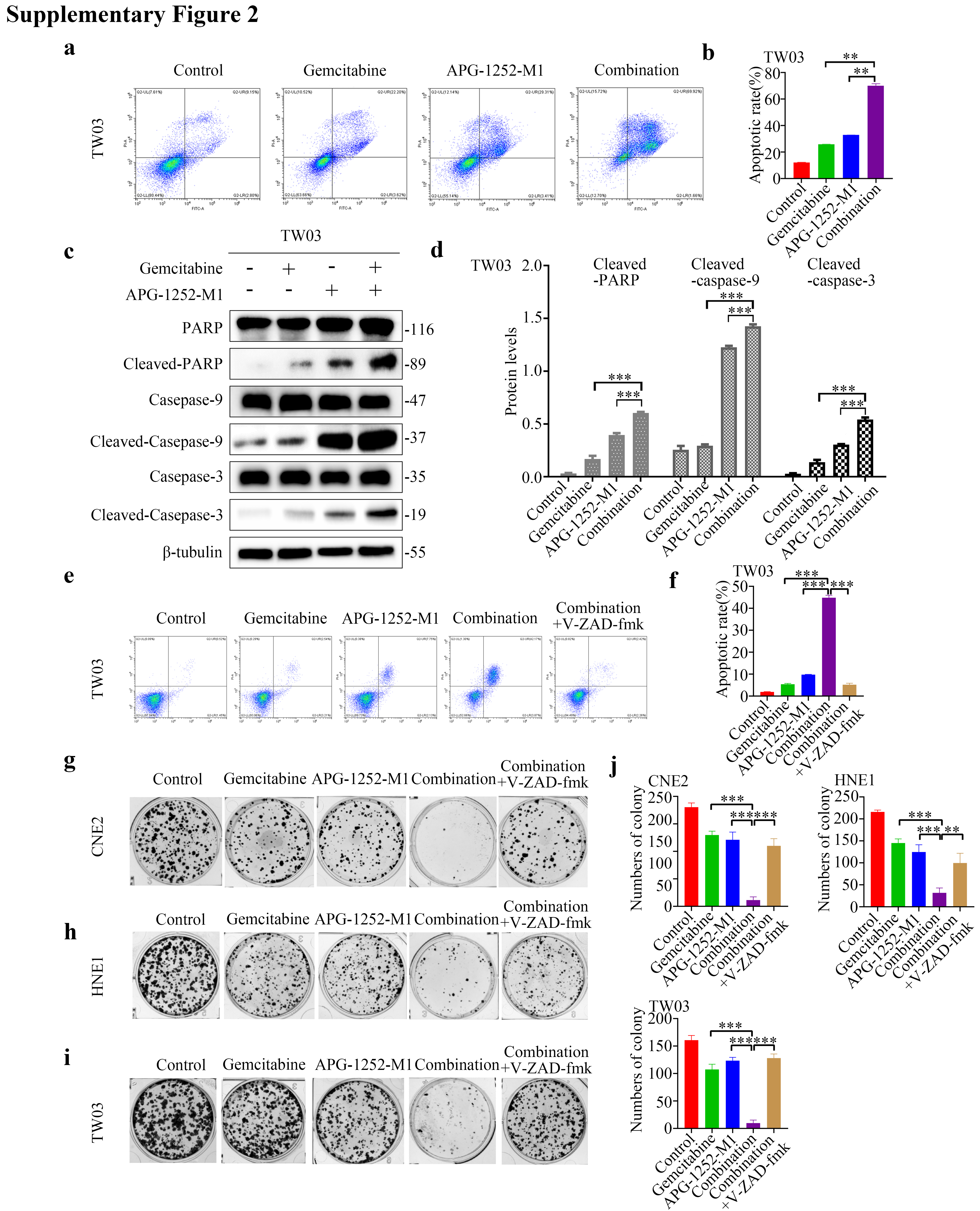

Supplement: Supplementary file 4 — Supplementary Figure 2 [file 41419_2021_4042_MOESM4_ESM.jpg]

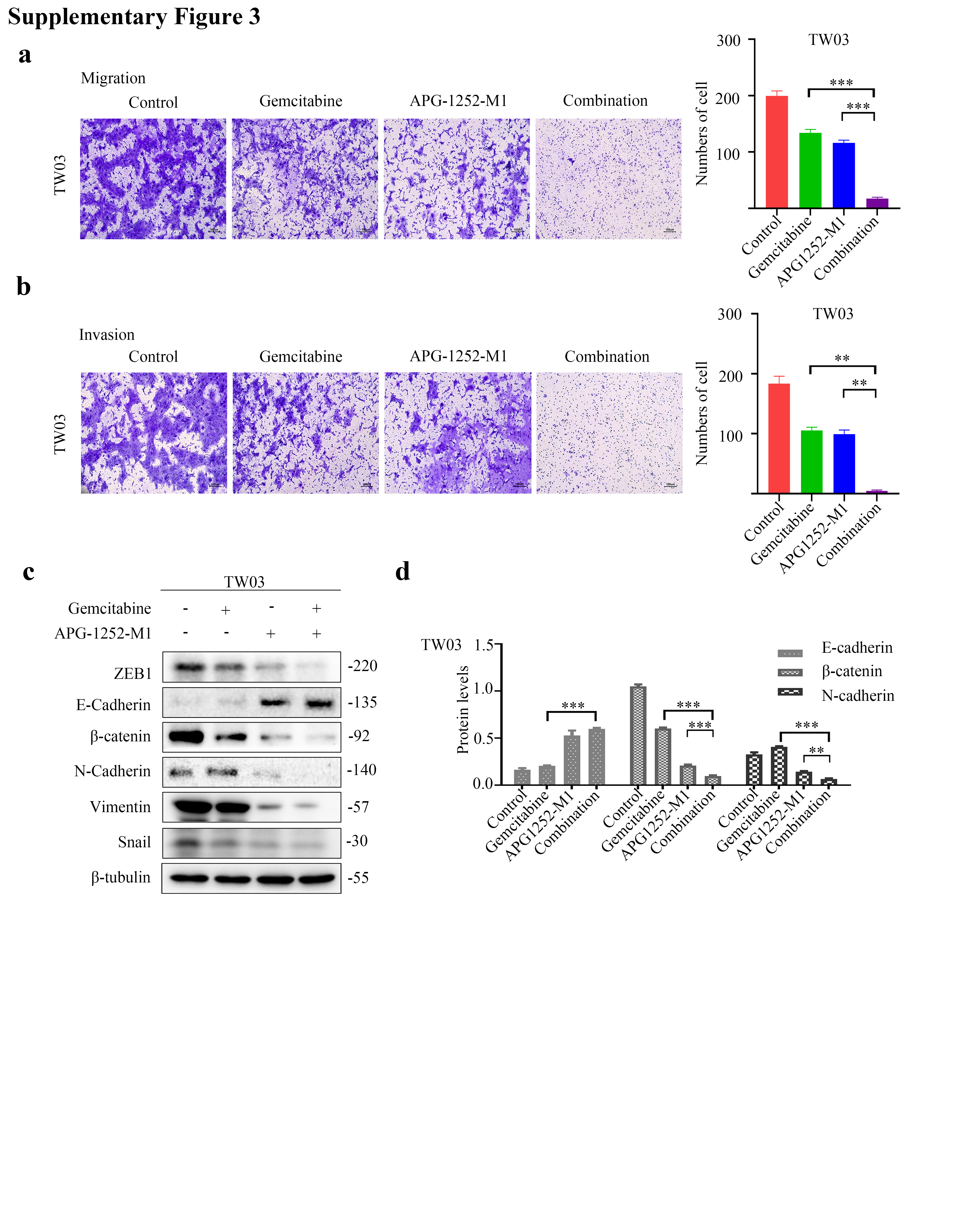

Supplement: Supplementary file 5 — Supplementary Figure 3 [file 41419_2021_4042_MOESM5_ESM.jpg]

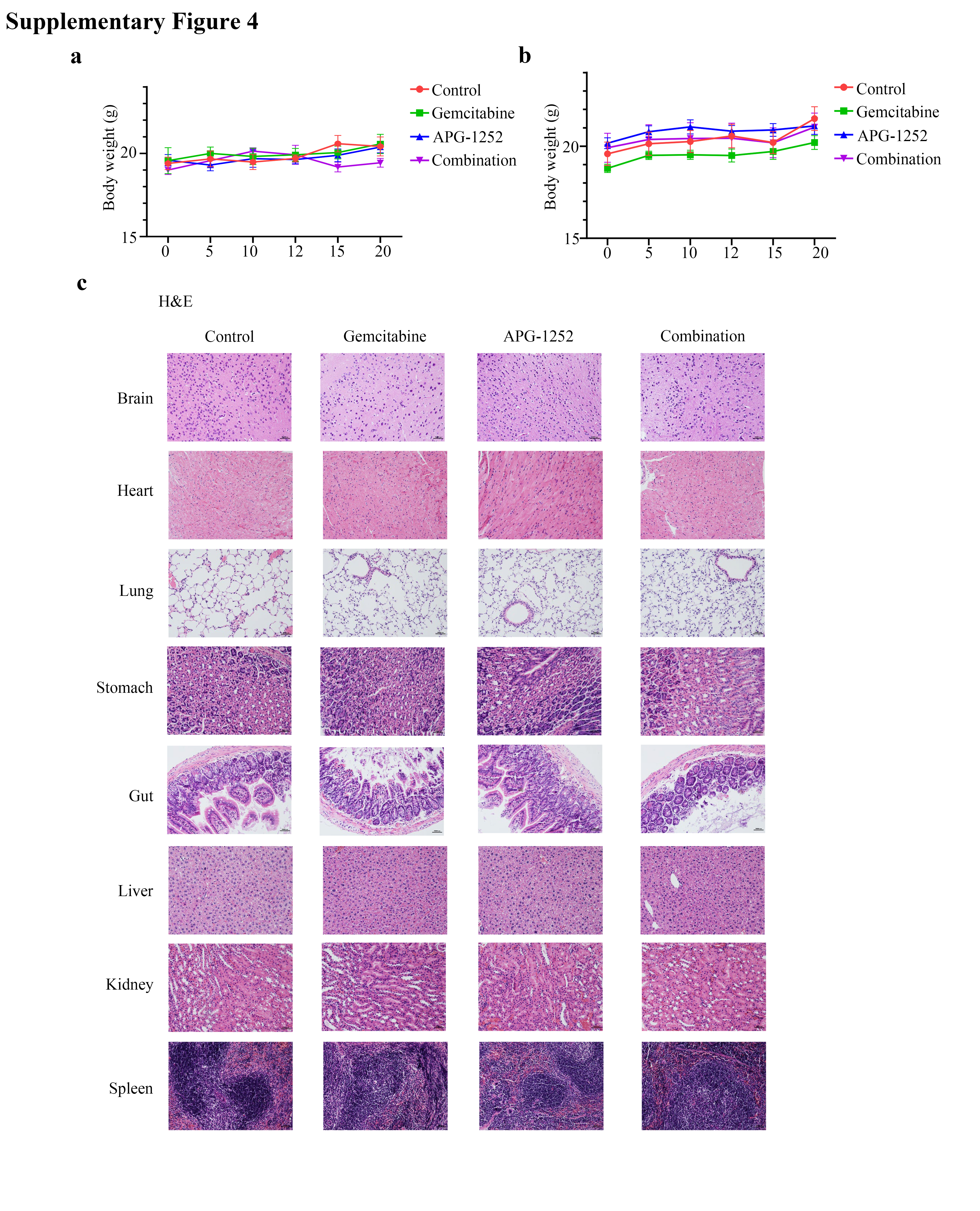

Supplement: Supplementary file 6 — Supplementary Figure 4 [file 41419_2021_4042_MOESM6_ESM.jpg]

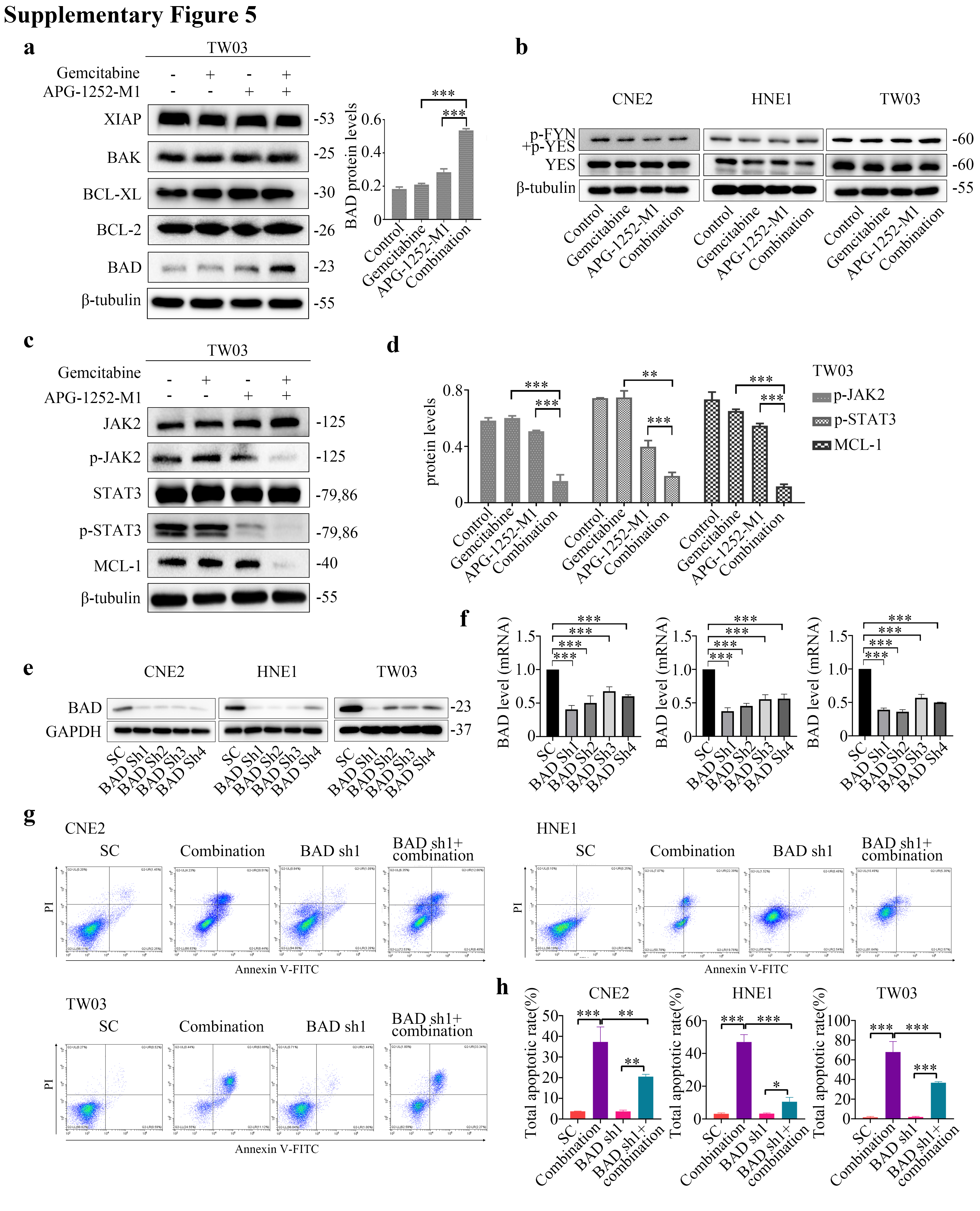

Supplement: Supplementary file 7 — Supplementary Figure 5 [file 41419_2021_4042_MOESM7_ESM.jpg]

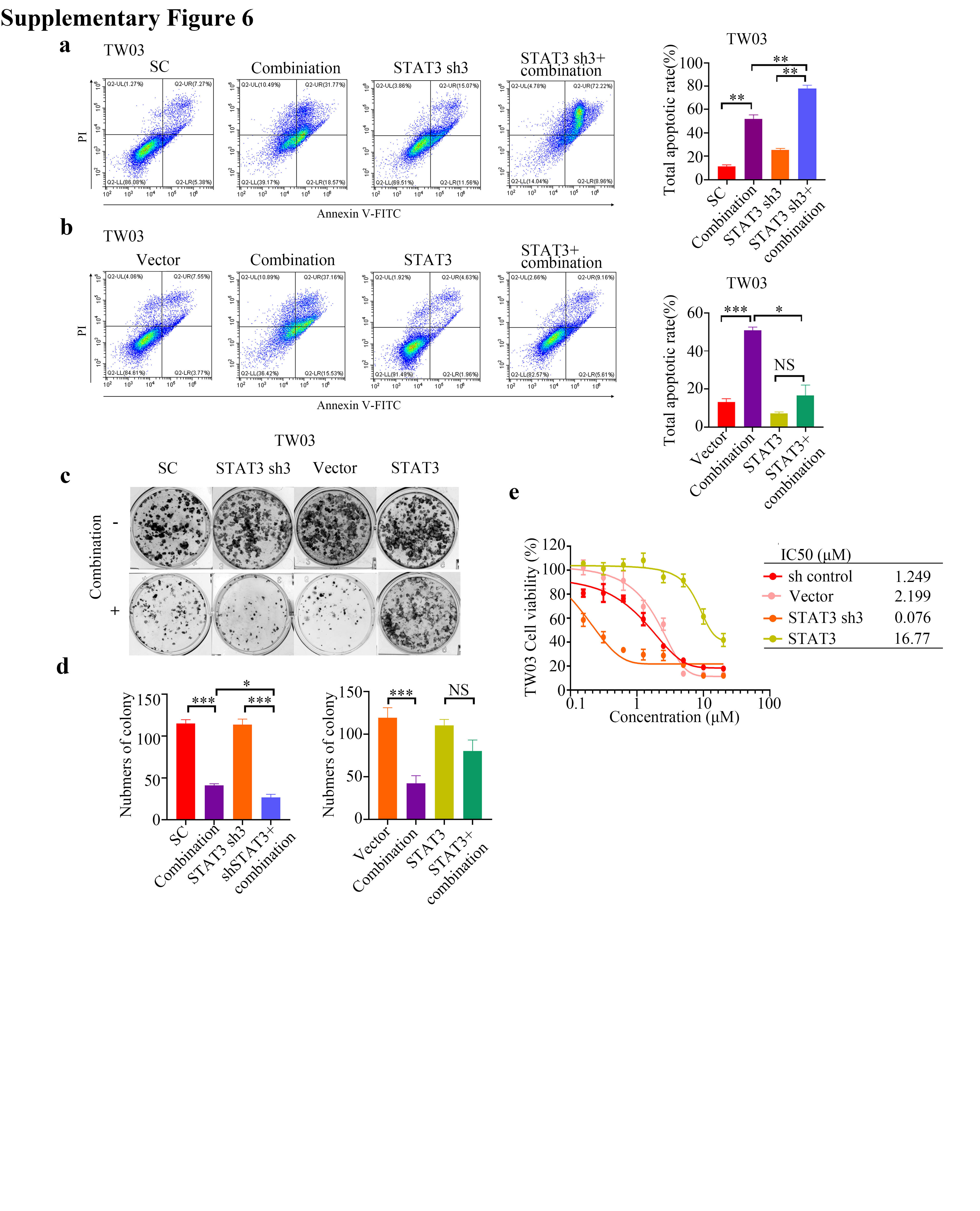

Supplement: Supplementary file 8 — Supplementary Figure 6 [file 41419_2021_4042_MOESM8_ESM.jpg]
